# Supplementary material for: circCDK13-loaded small extracellular vesicles accelerate healing in preclinical diabetic wound models
Source: Nat Commun. 2024 May 9;15:3904. doi: 10.1038/s41467-024-48284-3 (PMC11082226; doi:10.1038/s41467-024-48284-3)
Supplement: Supplementary file 1 — Supplementary Information [file 41467_2024_48284_MOESM1_ESM.pdf]

Supplementary Materials for

circCDK13-loaded small extracellular vesicles accelerates healing in preclinical diabetic wound models

Qilin Huang[1,2#], Ziqiang Chu[2,3#], Zihao Wang[2,3,4#], Qiankun Li[5], Sheng Meng[2,3], Yao Lu[5], Kui Ma[2,3], Shengnan Cui[2,6], Wenzhi Hu[2], Wenhua Zhang[2], Qian Wei[2], Yanlin Qu[2], Haihong Li[7*], Xiaobing Fu[2,3,8,9*], and Cuiping Zhang[2,3,8*]

#These authors contributed equally: Qilin Huang, Ziqiang Chu, and Zihao Wang

*Corresponding authors:

Haihong Li, MD, PhD; E-mail: lihaihong1051@126.com

Xiaobing Fu, MD, PhD; E-mail: fuxiaobing@vip.sina.com

Cuiping Zhang, MD, PhD; E-mail: zcp666666@sohu.com

This PDF file includes:

Supplementary figure 1 to Supplementary figure 18

Supplementary table 1 to Supplementary table 4

Supplementary Figures

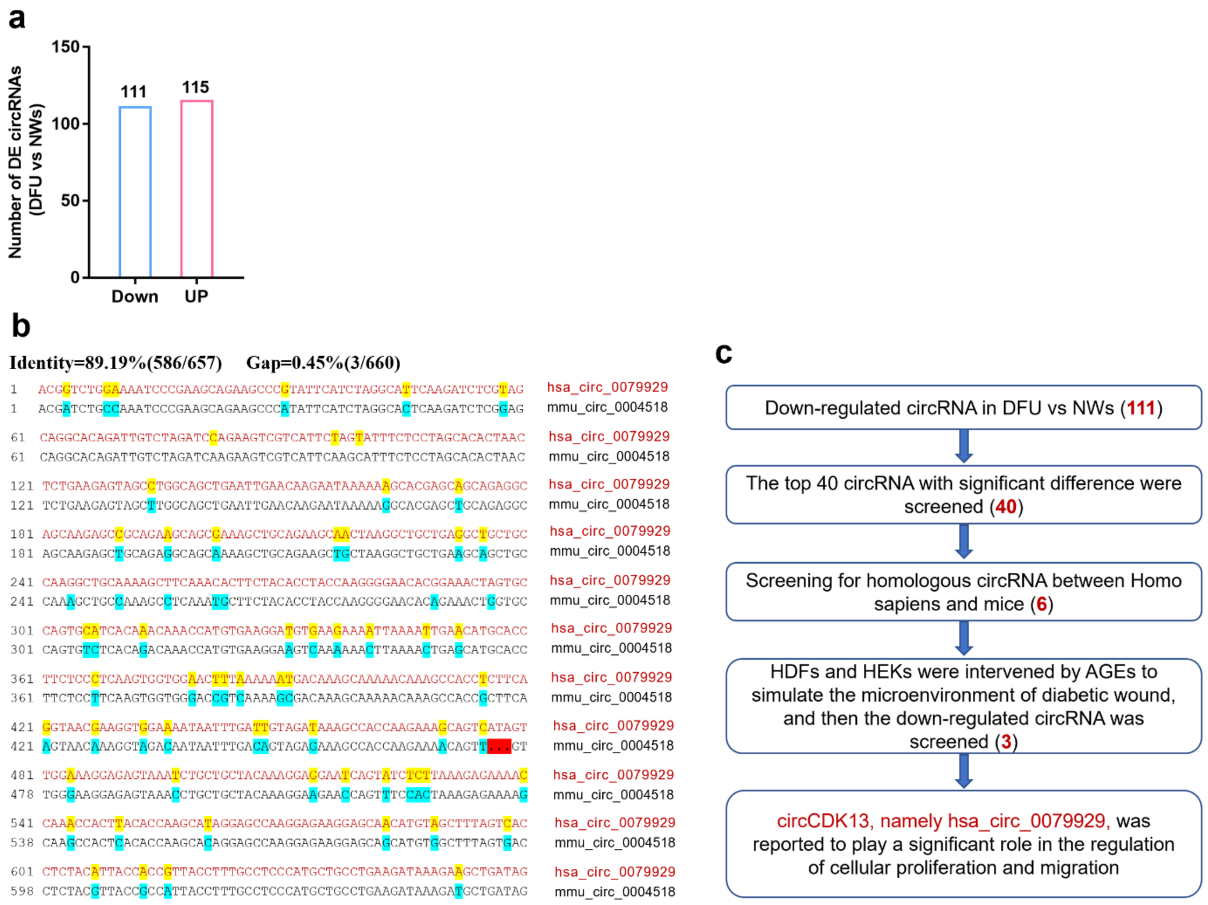

Supplementary Figure 1. Screening of circRNAs that promote wound healing

(a) Microarray analysis identified 115 upregulated and 111 downregulated circRNAs in the DFUs compared with the NWs [absolute fold-change (FC) ≥2, false discovery rate (FDR) <0.001, average raw intensity >100]. **(b)** Homology analysis of circCDK13 nucleic acid sequence between Homo sapiens (hsa_circ_0079929) and mice (mmu_circ_0004518). The nucleic acid sequence similarity between hsa_circ_0079929 and mmu_circ_0004518 is as high as 89.19%, indicating that circCDK13 is highly conserved between Homo sapiens and mice. **(c)** Schematic diagram of the circCDK13 screening process.

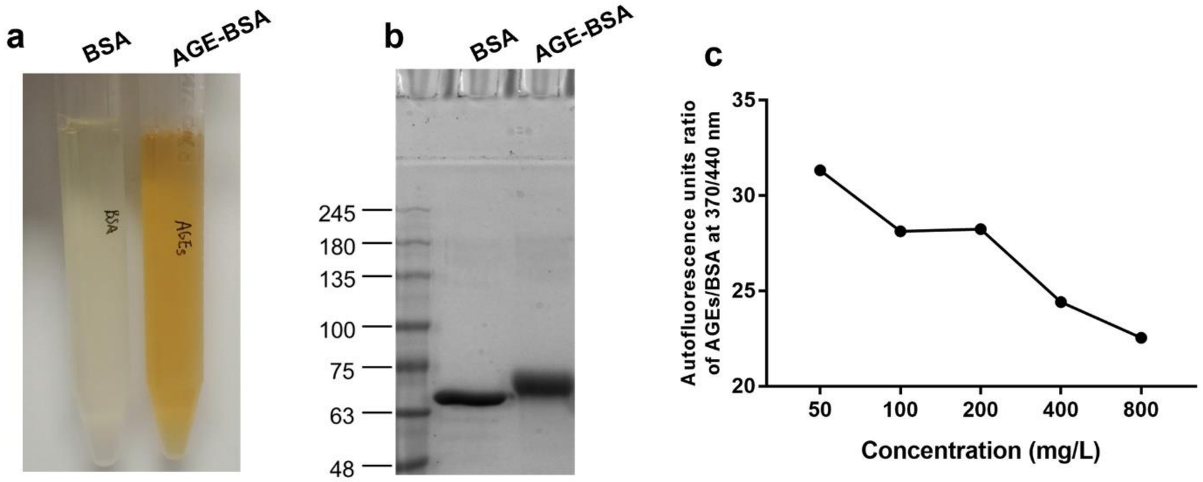

Supplementary Figure 2. Identification of AGE-BSA

(a) After incubation at 37°C in the dark for 2 months, the color of the product in the AGE-BSA group was significantly darker than that in the control group (BSA). **(b)** SDS-PAGE electrophoresis showed that the molecular weight of AGE-BSA was larger than that of BSA. **(c)** The autofluorescence intensity (λ_{ex}370 nm/λ_{em}440 nm) measured by fluorescence spectrometer showed that the fluorescence intensity of AGE-BSA was significantly higher than that of BSA with the same concentration.

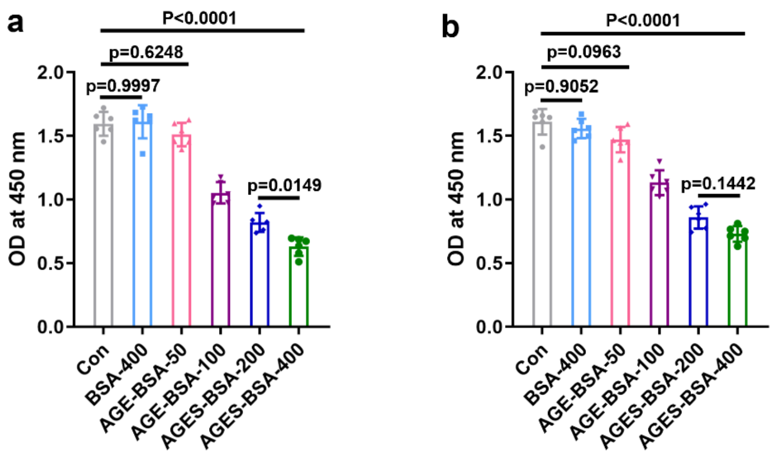

Supplementary Figure 3. AGE-BSA inhibits the proliferation of HDFs and HEKs in a concentration-dependent manner

(a) CCK-8 assay showed the proliferative ability of HDFs treated with different concentrations of AGE-BSA (n=6 biologically independent samples). **(b)** CCK-8 assay showed the proliferative ability of HEKs treated with different concentrations of AGE-BSA (n=6 biologically independent samples). Data are presented as mean values ± SD. Statistical significance and P values are analyzed by one-way ANOVA followed by Tukey’s multiple comparison test in **a, b**. Source data are provided as a Source Data file.

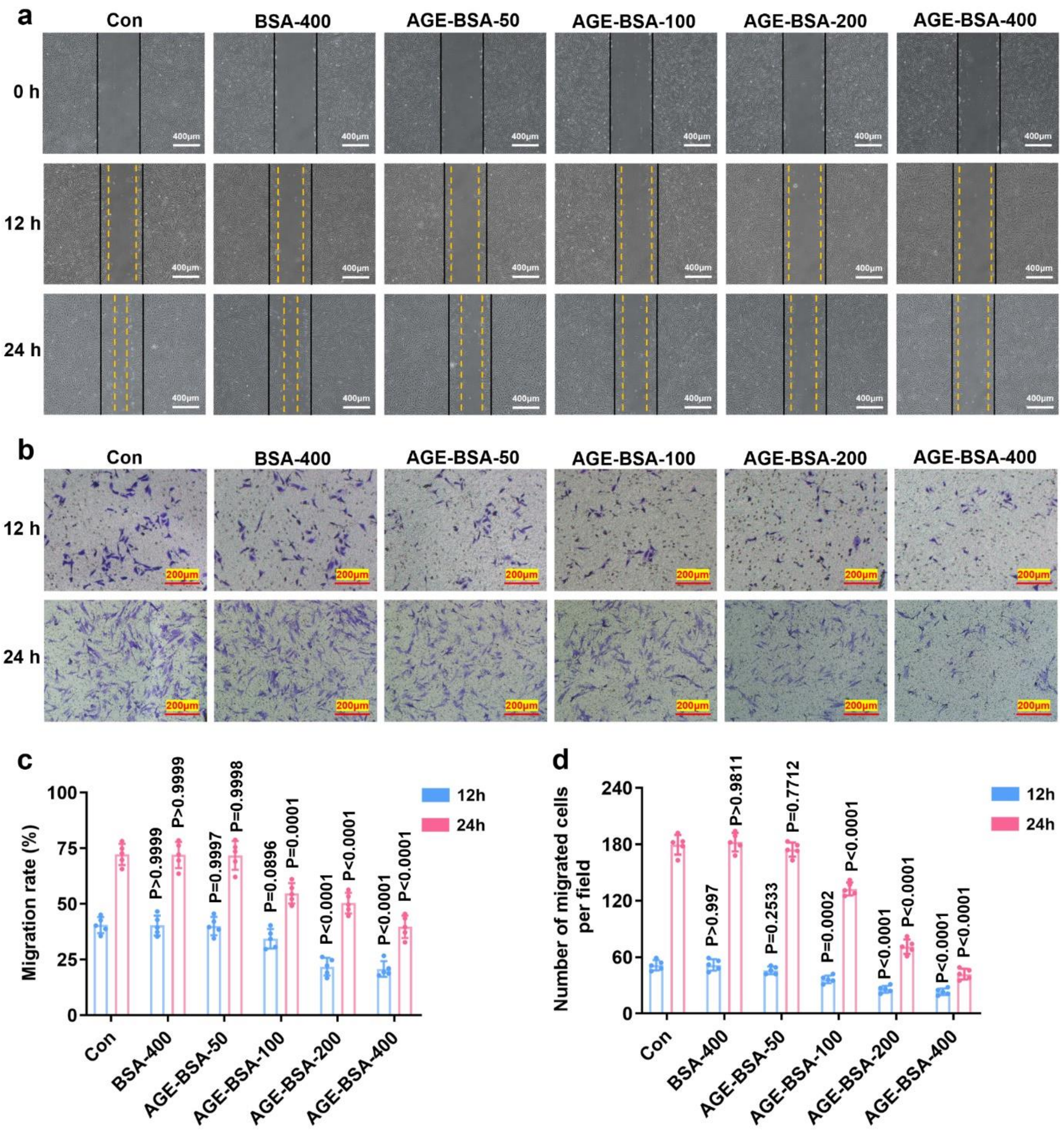

Supplementary Figure 4. AGE-BSA inhibits migration of HDFs

(a) Representative images of HDFs migration measured by wound healing assay at 0 h, 12 h, and 24 h. scale bar, 400 μm. **(b)** Representative images of transwell migration assay. scale bar, 200 μm. HDFs were treated with various concentrations of AGE-BSA (0 μg/ml, 50 μg/ml, 100 μg/ml, 200 μg/ml, and 400 μg/ml) or BSA (400 μg/ml). **(c)** Quantification of the mobility rate of HDFs in wound healing assay (n=5 biologically independent samples). **(d)** The number of migrated cells in transwell migration assay (n=5 biologically independent

samples). Data are presented as mean values ± SD. Statistical significance and P values are analyzed by one-way ANOVA followed by Dunnett's multiple comparison test in **c, d**. Source data are provided as a Source Data file.

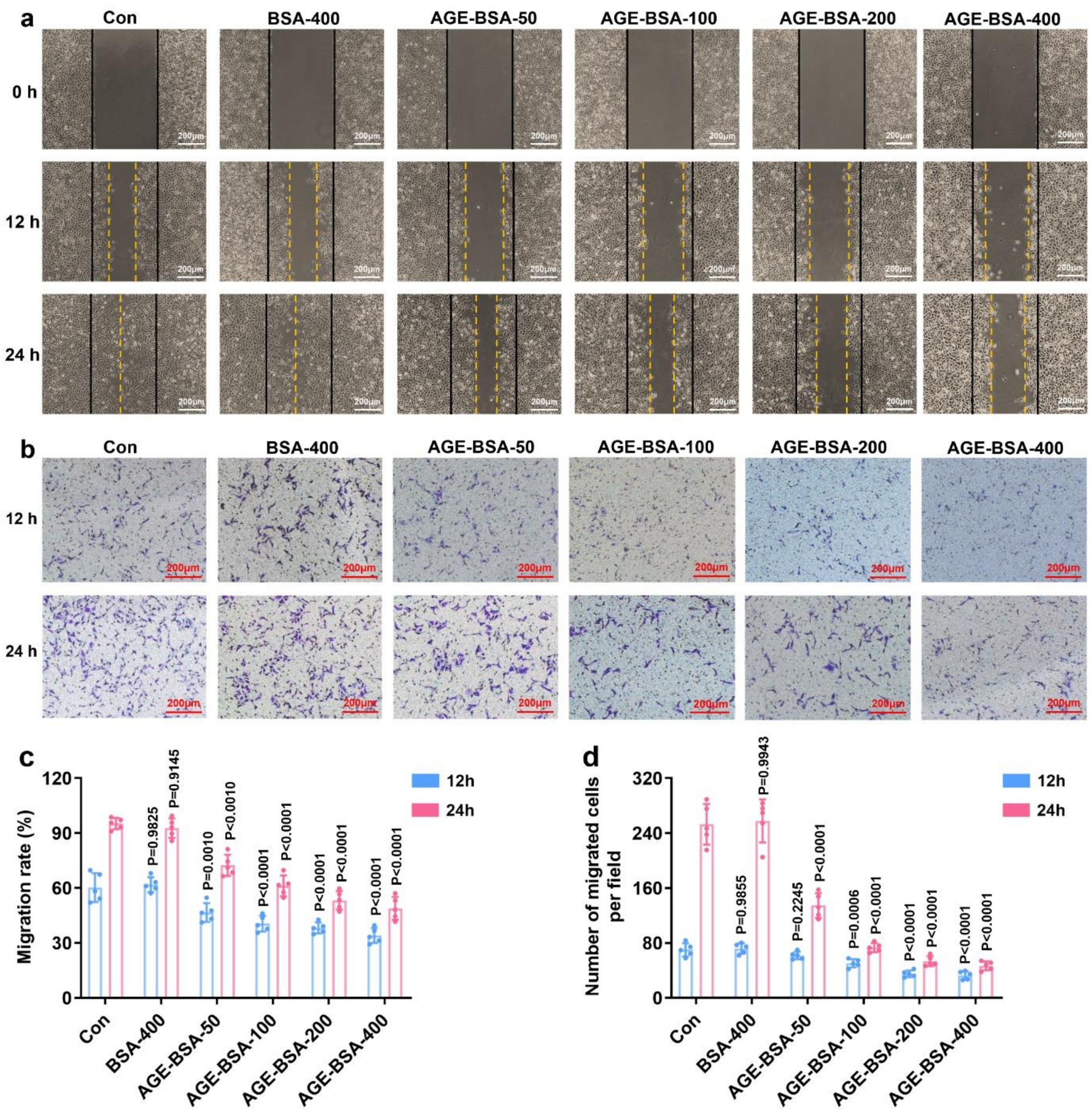

Supplementary Figure 5. AGE-BSA inhibits migration of HEKs

(a) Representative images of HEKs migration measured by wound healing assay at 0 h, 12 h, and 24 h. scale bar, 200 μm. **(b)** Representative images of transwell migration assay. scale bar, 200 μm. HEKs were treated with various concentrations of AGE-BSA (0 μg/ml, 50 μg/ml, 100 μg/ml, 200 μg/ml, and 400 μg/ml) or BSA (400 μg/ml). **(c)** Quantification of the mobility rate of HEKs in wound healing assay (n=5 biologically independent samples). **(d)** The number of migrated cells in transwell migration assay (n=5 biologically independent

samples). Data are presented as mean values ± SD. Statistical significance and P values are analyzed by one-way ANOVA followed by Dunnett's multiple comparison test in **c, d**. Source data are provided as a Source Data file.

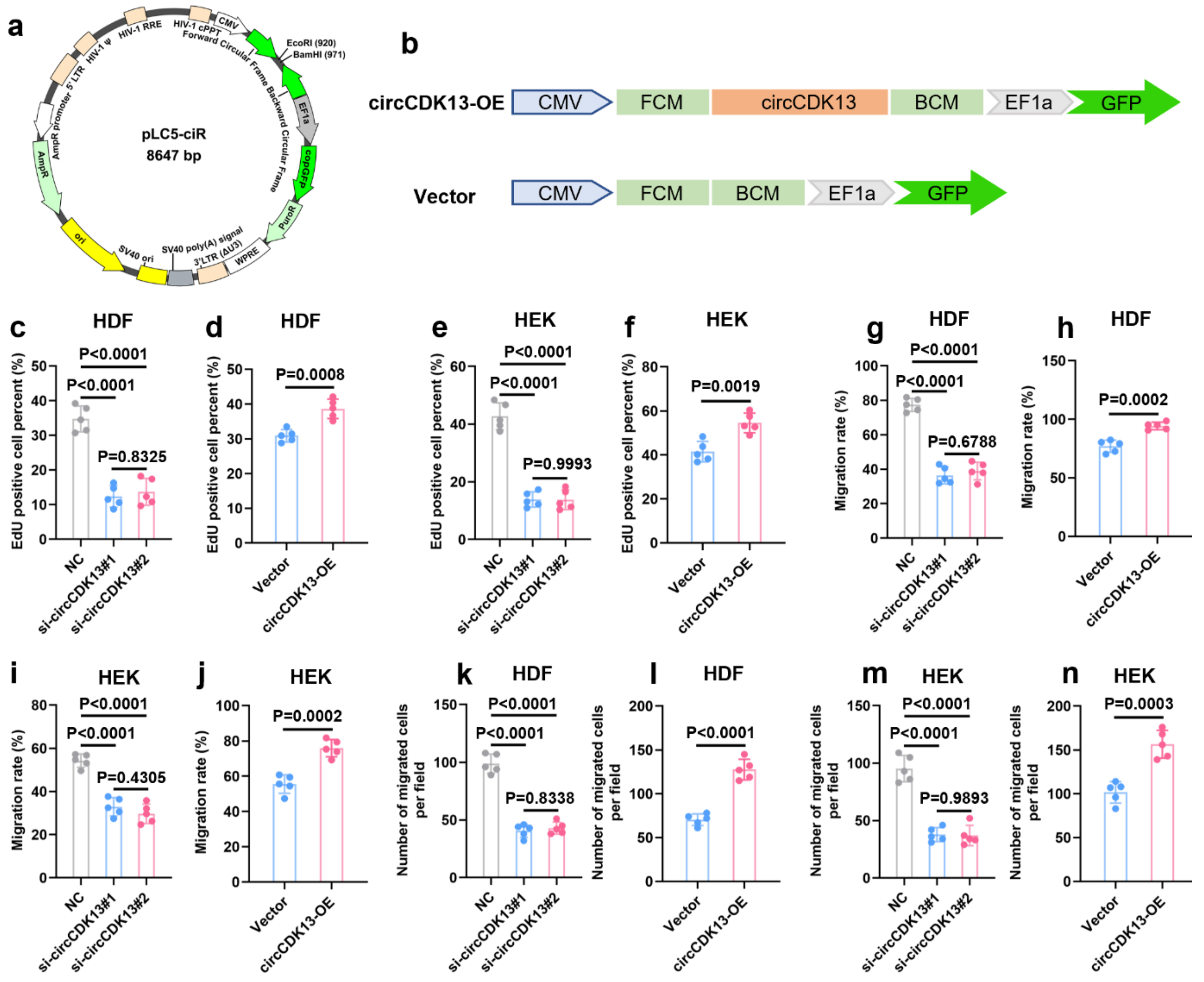

Supplementary Figure 6. circCDK13 promotes the proliferation and migration of HDFs and HEKs

(a) Schematic diagram of the plasmid structure used to construct the circCDK13 overexpression system. The pLC5-ciR plasmid was provided by Geneseed Biotechnology Co., Ltd (Guangzhou, China). **(b)** Structures of the circCDK13 expression construct and the control vector. **(c-f)** Quantification histogram represented EdU positive cell percentage (n=5 biologically independent samples). **(g-j)** Quantification of the mobility rate of HDFs and HEKs in wound healing assay (n=5 biologically independent samples). **(k-n)** The number of migrated cells in transwell migration assay (n=5 biologically independent samples). Comparisons were performed by one-way ANOVA followed by Tukey's multiple

comparisons test in (**c, e, g, i, k, and m**) and two-tailed Student's t test in (**d, f, h, j, l, and n**).

Data are presented as mean values ± SD. Source data are provided as a Source Data file.

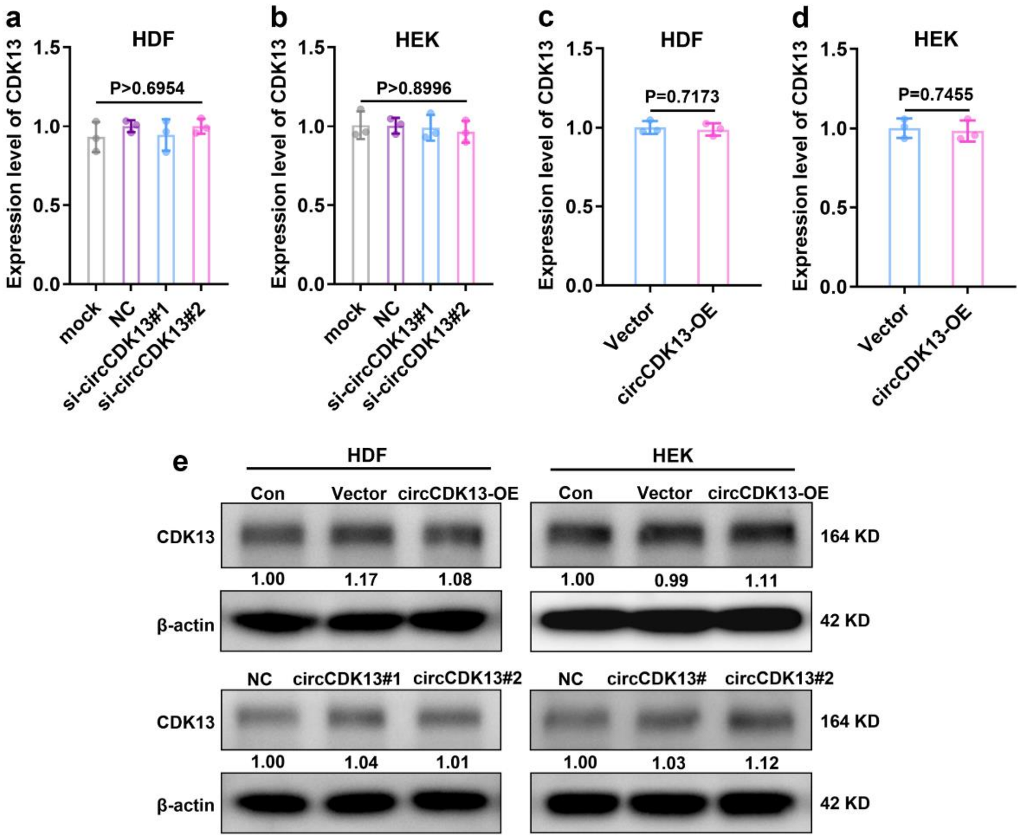

Supplementary Figure 7. CDK13 mRNA and protein expression in HDFs and HEKs is unaffected by circCDK13 expression

(a, b) Knockdown of circCDK13 by siRNA interference technology in HDFs and HEKs does not affect the expression level of CDK13 mRNA (n=3 biologically independent samples). **(c, d)** Exogenous enforced overexpression of circCDK13 in HDFs and HEKs fails to alter the expression level of CDK13 mRNA (n=3 biologically independent samples). **(e)** The expression of circCDK13 was overexpressed or knocked down in HDFs and HEKs, and then the expression level of CDK13 protein in cells was detected by western blot. Three independent experiments were carried out with similar results. Comparisons were performed by one-way ANOVA followed by Tukey's multiple comparisons test in (**a, b**) and two-tailed Student's t test in (**c, d**). Data are presented as mean values ± SD. Source data are provided as a Source Data file.

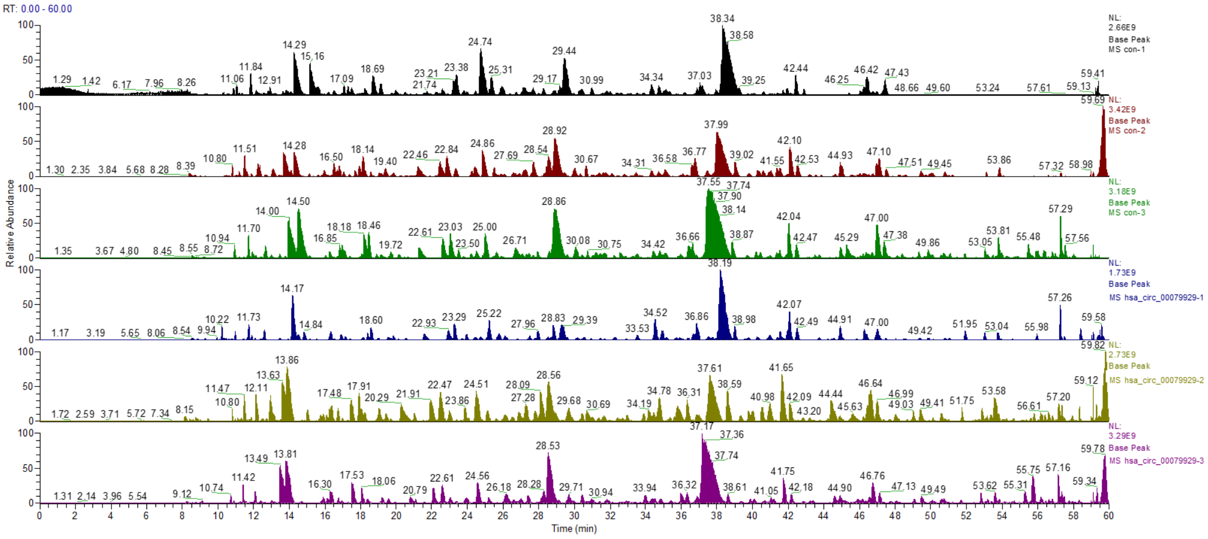

Supplementary Figure 8. Base peak plot of mass spectrometry

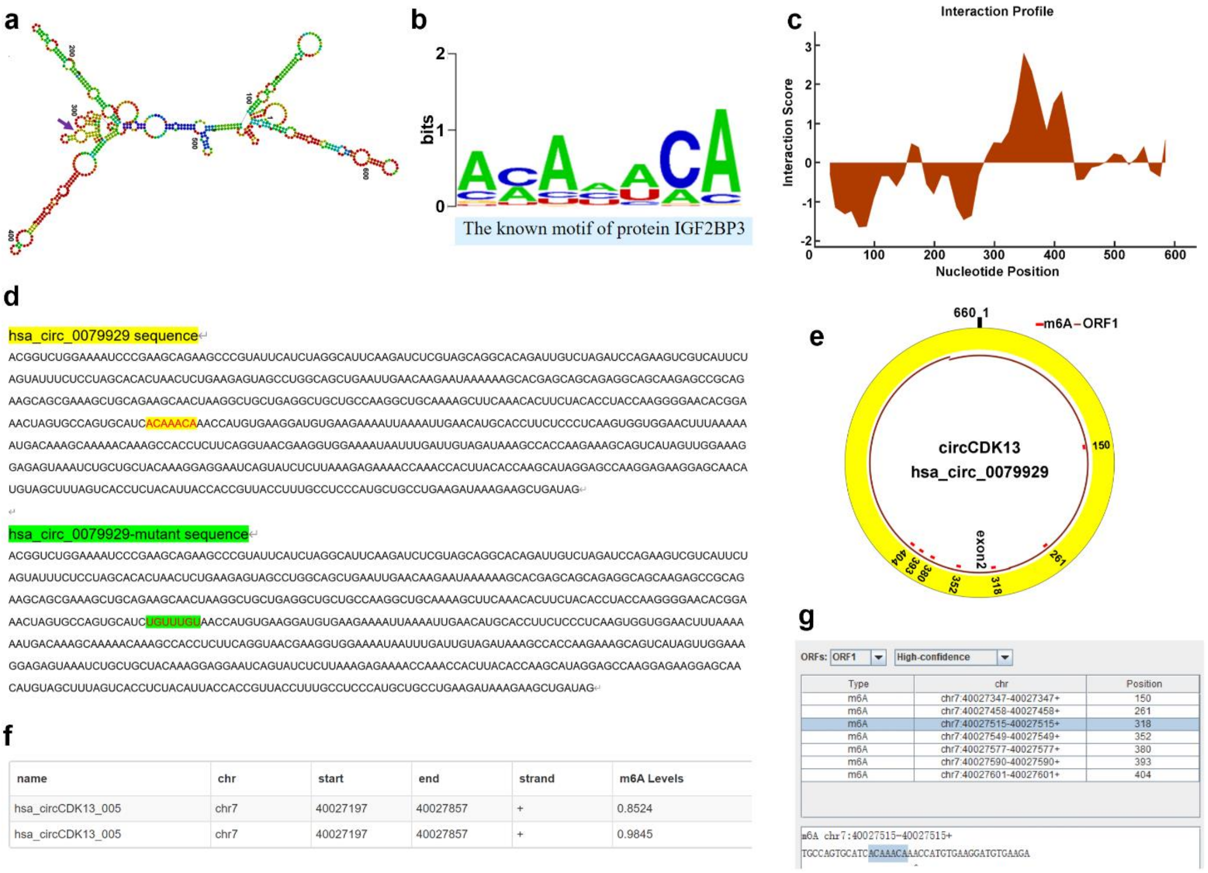

Supplementary Figure 9. Prediction of binding sites between circCDK13 and IGF2BP3

(a) The secondary structure of circCDK13 was predicted by RNAfold (http://rna.tbi.univie.ac.at/cgi-bin/RNAWebSuite/RNAfold.cgi). **(b)** The binding motif of IGF2BP3 and circCDK13 was predicted by RBP suite (http://www.csbio.sjtu.edu.cn/bioinf/RBPsuite/). **(c)** Binding sites of circCDK13 and IGF2BP3 predicted by catRAPID fragments (http://service.tartaglialab.com/page/catrapid_omics2_group). **(d)** Sequences of circCDK13 and circCDK13-mutant. For the circCDK13-mutant plasmid, “ACAAACA” was replaced with “UGUUUGU”. **(e)** The m^6A-modified site that might exist in circCDK13 predicted by circPrimer 2.0 software. **(f)** Prediction of circCDK13 m^6A modification levels by circBank online tool (http://www.circbank.cn/). **(g)** Analysis of circCDK13 m^6A modification sites by circPrimer 2.0 software.

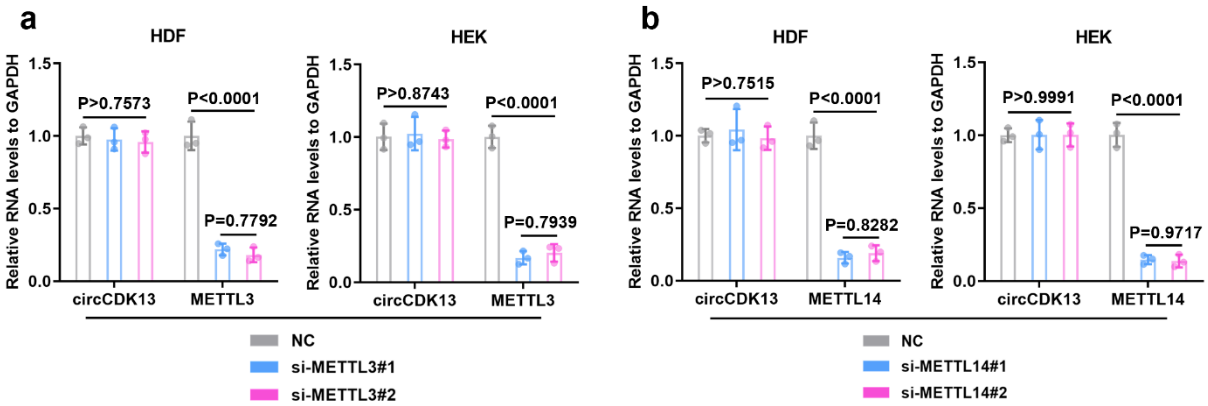

Supplementary Figure 10. Effect of METTL3/14 siRNAs on circCDK13

(a) After knocking down METTL3 in HDFs and HEKs, the expression levels of circCDK13 and METTL3 mRNA were detected by RT-qPCR assay. **(b)** After knocking down METTL14 in HDFs and HEKs, the expression levels of circCDK13 and METTL14 mRNA were detected by RT-qPCR assay. Comparisons were performed by one-way ANOVA followed by Tukey's multiple comparisons test in (**a, b**). Data are presented as mean values ± SD (n=3 biologically independent samples). Source data are provided as a Source Data file.

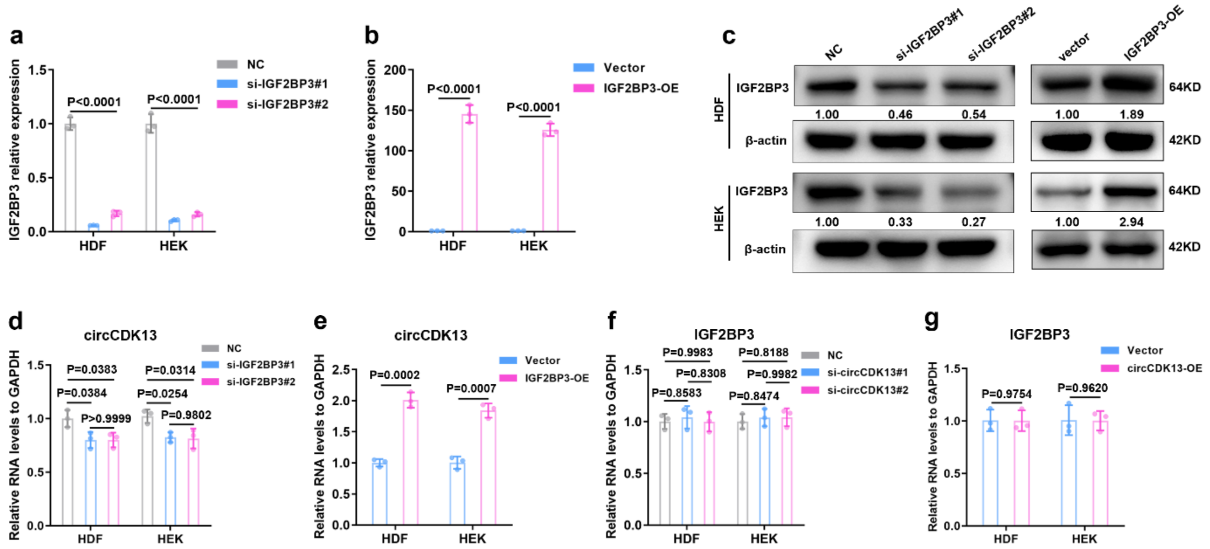

Supplementary Figure 11. Effect of IGF2BP3 on expression level of circCDK13

(a, b) The expression level of IGF2BP3 mRNA was detected by RT-qPCR assay after knockdown or overexpression of IGF2BP3 in HDFs and HEKs (n=3 biologically independent samples). **(c)** Western blot assay detected the IGF2BP3 expression in HDFs and HEKs transfected with IGF2BP3-OE, si-IGF2BP3#1, si-IGF2BP3#2 or their respective control vector. **(d, e)** Abundance of circCDK13 detected by RT-qPCR assay after knockdown or overexpression of IGF2BP3 in HDFs and HEKs (n=3 biologically independent samples). **(f, g)** After knockdown or overexpression of circCDK13 in HDFs and HEKs, the expression level of IGF2BP3 mRNA was detected by RT-qPCR assay (n=3 biologically independent samples). Comparisons were performed by one-way ANOVA followed by Tukey's multiple comparisons test in (**a, d, and f**) and two-tailed Student's t test in (**b, e, and g**). Data are presented as mean values ± SD. Three independent experiments were carried out with similar results in **c**. Source data are provided as a Source Data file.

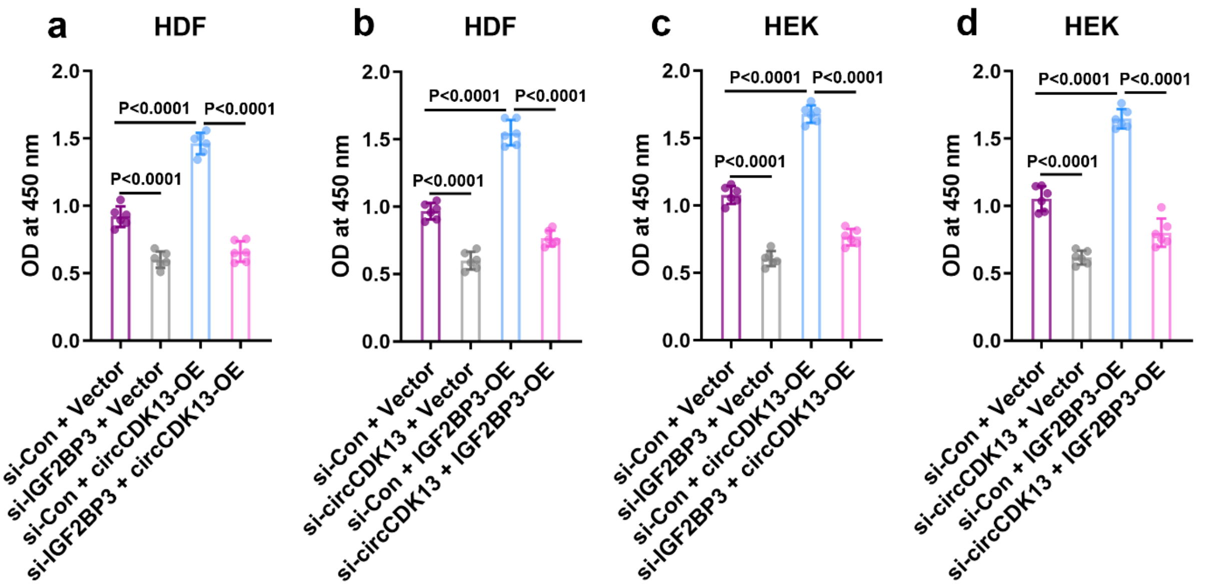

Supplementary Figure 12. circCDK13 cooperates with IGF2BP3 to promote the proliferation of HDFs and HEKs

(a) CCK-8 assay showed that IGF2BP3 knockdown abolished the induction of HDFs proliferation by the circCDK13 overexpression. **(b)** CCK-8 assay showed that circCDK13 knockdown abolished the induction of HDFs proliferation by the IGF2BP3 overexpression. **(c)** CCK-8 assay showed that IGF2BP3 knockdown abrogated the promoting effects of circCDK13 overexpression on HEKs proliferation. **(d)** CCK-8 assay showed that circCDK13 knockdown abrogated the promoting effects of IGF2BP3 overexpression on HEKs proliferation. Comparisons were performed by one-way ANOVA followed by Tukey's multiple comparisons test in (**a, b, c, and d**). Data are presented as mean values ± SD (n=6 biologically independent samples). Source data are provided as a Source Data file.

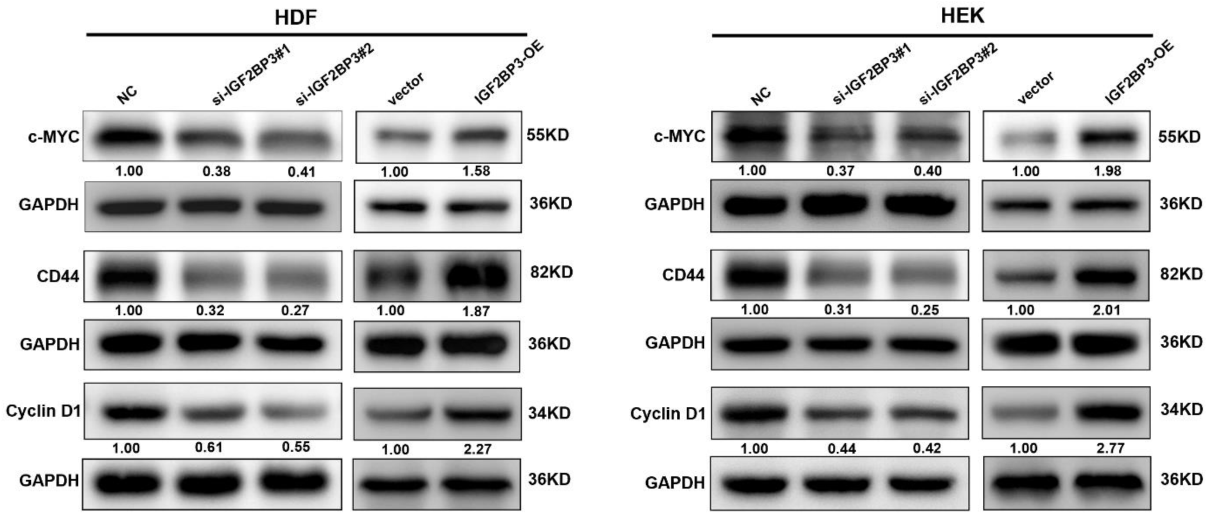

Supplementary Figure 13. Effects of knockdown or overexpression of IGF2BP3 on c-MYC, CD44, and Cyclin D1

The protein levels of c-MYC, CD44, and Cyclin D1 were measured by western blot assay in HDFs and HEKs transfected with si-NC or si-IGF2BP3, and vector or IGF2BP3-OE. Three independent experiments were carried out with similar results. Source data are provided as a Source Data file.

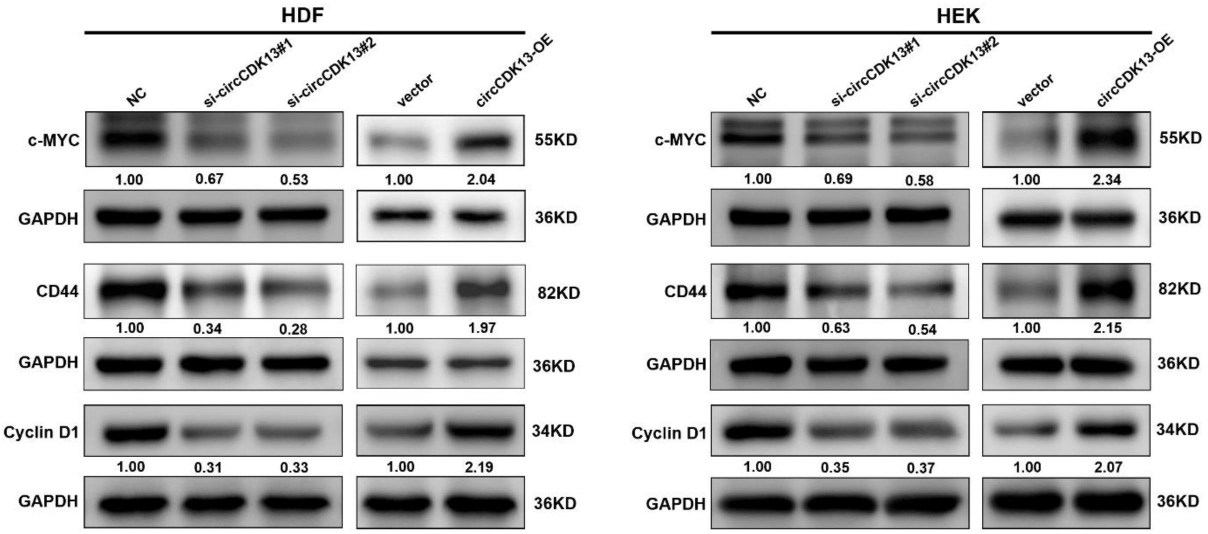

Supplementary Figure 14. Effects of knockdown or overexpression of circCDK13 on c-MYC, CD44, and Cyclin D1

The protein levels of c-MYC, CD44, and Cyclin D1 were detected by western blot assay in HDFs and HEKs transfected with si-NC or si-circCDK13, and vector or circCDK13-OE. Three independent experiments were carried out with similar results. Source data are provided as a Source Data file.

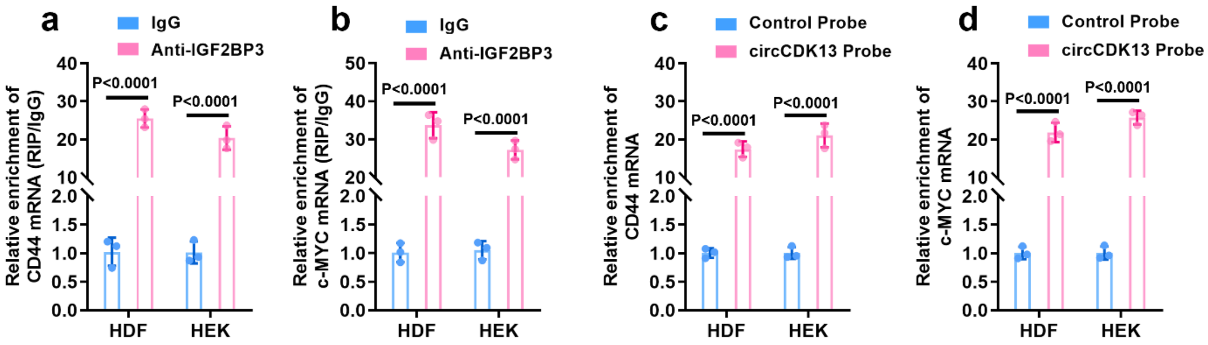

Supplementary Figure 15. PCR analysis of pull-down products of circCDK13 probe or IGF2BP3 antibody in HDFs and HEKs

(a) RT-qPCR analysis of relative enrichment of CD44 mRNA in IGF2BP3 pull-down products. **(b)** RT-qPCR analysis of relative enrichment of c-MYC mRNA in IGF2BP3 pull-down products. **(c)** RT-qPCR analysis of relative enrichment of CD44 mRNA in circCDK13 pull-down products. **(d)** RT-qPCR analysis of relative enrichment of c-MYC mRNA in circCDK13 pull-down products. Comparisons were performed by two-tailed Student's t test in (**a**, **b**, **c**, **and d**). Data are presented as mean values ± SD (n=3 biologically independent samples). Source data are provided as a Source Data file.

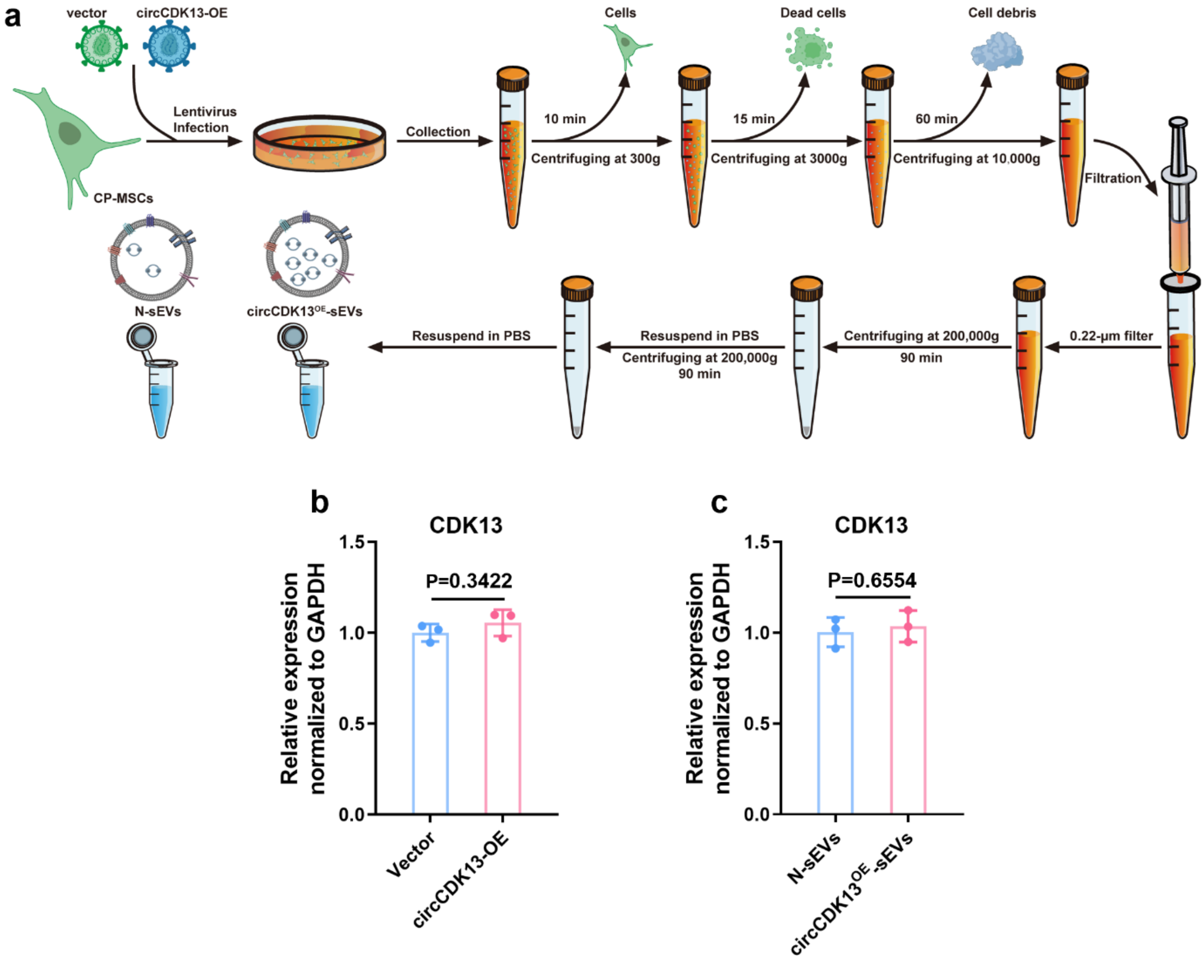

Supplementary Figure 16. Preparation and characterization of engineered sEVs

(a) Schematic diagram of the production and harvest of engineered sEVs bearing circCDK13. **(b)** RT-qPCR analysis of CDK13 mRNA abundance in CP-MSCs transduced with vector or circCDK13 lentivirus. **(c)** RT-qPCR analysis of CDK13 mRNA abundance in N-sEVs and circCDK13^{OE}-sEVs. Comparisons were performed by two-tailed Student's t test in (**b and c**). Data are presented as mean values ± SD (n=3 biologically independent samples). Source data are provided as a Source Data file.

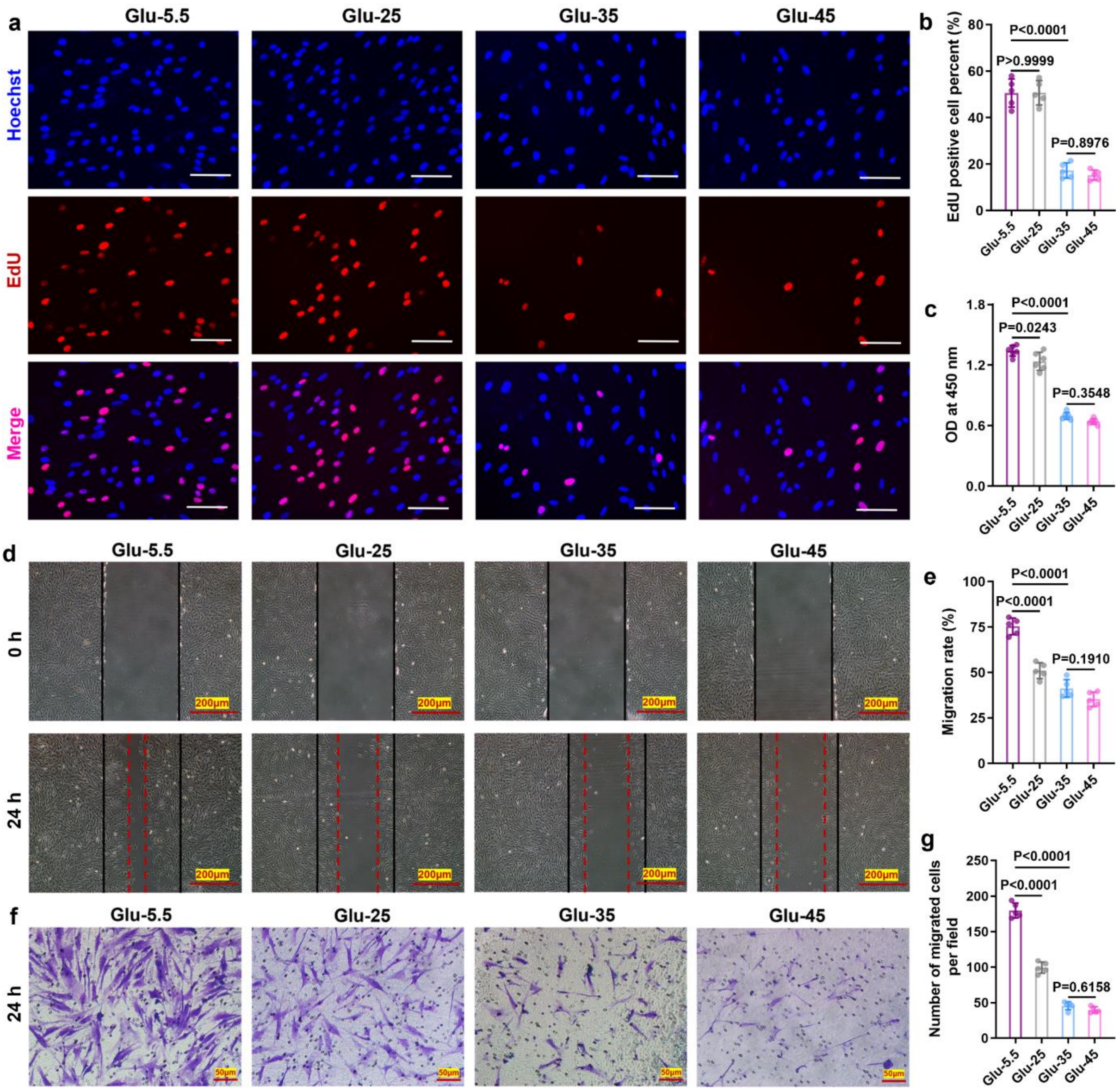

Supplementary Figure 17. High-glucose inhibits the proliferation and migration of HDFs

(a) EdU incorporation assay was performed to assess DNA synthesis in HDFs. scale bar, 100 μm. **(b)** Quantification histogram represented EdU positive cell percentage (n=5 biologically independent samples). **(c)** CCK-8 assay was performed to detect the proliferation potential of HDFs (n=6 biologically independent samples). **(d)** Wound healing assay was performed to measure the motility of HDFs. scale bar, 200 μm. **(e)** Quantification histogram represented migration rate of HDFs (n=5 biologically independent samples). **(f)** Representative images of

transwell migration assay of HDFs. scale bar, 50 μm. **(g)** Quantification histogram represented the number of migrated HDFs (n=5 biologically independent samples). HDFs were treated with different concentrations of D-glucose (D-glucose concentration, 5.5 mM, 25 mM, 35 mM, and 45 mM). Comparisons were performed by one-way ANOVA followed by Tukey's multiple comparisons test in (**b, c, e, and g**). Data are presented as mean values ± SD. Source data are provided as a Source Data file.

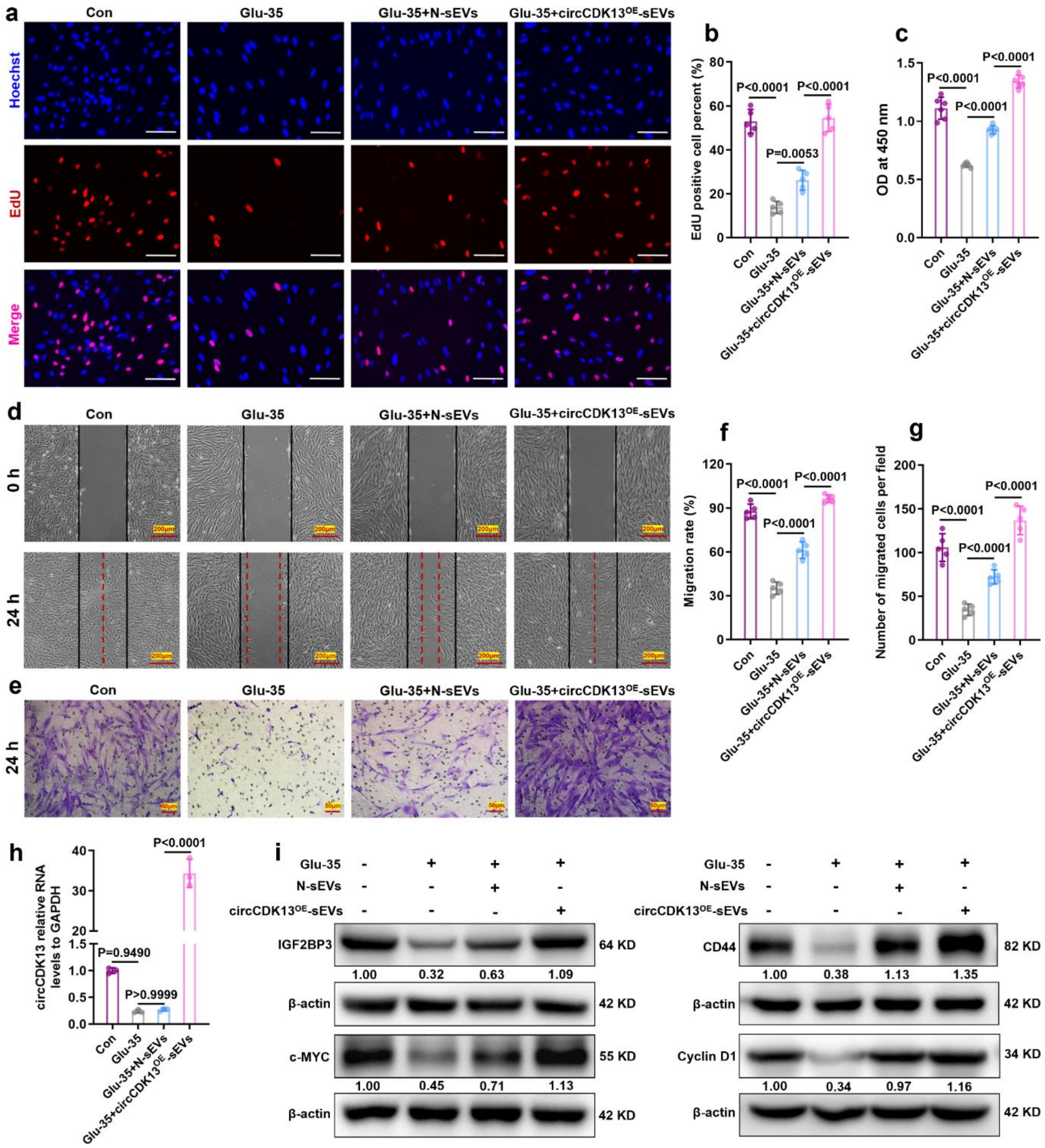

Supplementary Figure 18. circCDK13^{OE}-sEVs abolish the inhibitory effect of high-glucose on HDFs proliferation and migration

(a) Cell proliferation was evaluated with an EdU incorporation assay in HDFs. scale bar, 100 μm. **(b)** Quantification histogram represented EdU positive cell percentage (n=5 biologically independent samples). **(c)** The CCK-8 assay was used to evaluate the proliferative ability of HDFs (n=6 biologically independent samples). **(d)** Wound healing assay was performed to

measure the motility of HDFs. scale bar, 200 μm. **(e)** Transwell migration assay was performed to measure the motility of HDFs. scale bar, 50 μm. **(f)** Quantitative analysis of migration rate in the wound healing assay of HDFs (n=5 biologically independent samples). **(g)** Quantitative analysis of the number of migrated cells in transwell migration assay of HDFs (n=5 biologically independent samples). (**h**) RT-qPCR analysis of circCDK13 abundance in HDF after co-incubation with N-sEVs or circCDK13^{OE}-sEVs for 6 h (n=3 biologically independent samples). (**i**) Western blots analysis of IGF2BP3, CD44, c-MYC, and cyclin D1 protein expression levels in HDFs of different treatment groups. sEVs concentration, 2×10^{10} particles/ml. Comparisons were performed by one-way ANOVA followed by Tukey's multiple comparisons test in (**b, c, f, g, and h**). Data are presented as mean values ± SD. Three independent experiments were carried out with similar results in **i**. Source data are provided as a Source Data file.

Supplementary Tables

Supplementary Table 1. Primer used for real-time quantitative PCR (RT-qPCR)

mRNA	Forward sequence (5’-3’)	Reverse sequence (5’-3’)
circCDK13（Human）	GCCAAGGAGAAGGAGCAACAT	GAATACGGGCTTCTGCTTCG
circCDK13（mouse）	CCAAGGAGAAGGAGCAGCAT	CTCCGAGATCTTGAGTGCCT
CD44（Human）	GGCAAGAAACCTGGGATTGG	CTGAGGTGTCTGTCTCTTTCATC
c-MYC（Human）	GTCAAGAGGCGAACACACAAC	TTGGACGGACAGGATGTATGC
CDK13（Human）	CCCTGAGCTACCAGGAGGAGATG	CAGTGTCTTTATCCCTGGCTTTGT
IGF2BP3（Human）	ATCGGAAACCTCAGCGAGAA	TTTTACCTGAAAGCGCCTCG
GAPDH（Human）	AGAAGGCTGGGGCTCATTTG	GCAGGAGGCATTGCTGATGAT
U6（Human）	CTCGCTTCGGCAGCACA	AACGCTTCACGAATTTGCGT
METTL3（Human）	ACACCACCTCTCTGATCTGGCC	CTCCTGAGCTGCAAACTTCTGC
METTL14（Human）	ATGGATAGCCGCTTGCAGG	ACACGGCACCAATGCTGTCG
circCDK13-Divergent	TCACCTCTACATTACCACCGTTA	CTAGACAATCTGTGCCTGCTAC
circCDK13-Convergen	AAGAAAGCAGTCATAGTTGGAAAGG	AGGTAACGGTGGTAATGTAGAGG
GAPDH-Divergent	CATTGCCCTCAACGACCACT	ACCTTCACCTTCCCCATTTACT
GAPDH-Convergen	ACATCATCCCTGCCTCTACTG	CCTGCTTCACCACCTTCTTG

Supplementary Table 2. RNA Fluorescence in situ hybridization (FISH) probe sequence

	Targeted Sequence (5’-3’)
circCDK13-FISH probe	TTCGGGATTTTCCAGACCGTCTATCAGCTTCTTTATCTTC-CY3

Supplementary Table 3. The detailed siRNA sequences used in this study

	Targeted Sequence (5’-3’)
si-circCDK13#1	5’- AGCTGATAGACGGTCTGGA tt
si-circCDK13#2	5’- GCTGATAGACGGTCTGGAA tt
si-IGF2BP3#1	5’- GCAAAGGAUUCGGAAACUU tt
si-IGF2BP3#2	5’- GCUGCUGAGAAGUCGAUUA tt
si-METTL3#1	5’-GCUACCUGGACGUCAGUAU tt
si-METTL3#2	5’-GGUUGGUGUCAAAGGAAAU tt
si-METTL14#1	5’-GGAUGAAGGAGAGACAGAU tt
si-METTL14#2	5’-GCAGCACCUCGAUCAUUUA tt

Supplementary Table 4. Biotinylated RNA probes used for RNA pulldown

	Targeted Sequence (5’-3’)
circCDK13 sense	Biotin-TTCGGGATTTTCCAGACCGTCTATCAGCTTCTTTATCTTC
circCDK13 anti-sense	Biotin-GAAGATAAAGAAGCTGATAGACGGTCTGGAAAATCCCGAA
