# Supplementary material for: circCDK13-loaded small extracellular vesicles accelerate healing in preclinical diabetic wound models
Source: Nat Commun. 2024 May 9;15:3904. doi: 10.1038/s41467-024-48284-3 (PMC11082226; doi:10.1038/s41467-024-48284-3)
Supplement: Supplementary file 3 — Reporting summary [file 41467_2024_48284_MOESM3_ESM.pdf]

nature portfolio

Corresponding author(s): Haihong Li, Xiaobing Fu, and Cuiping Zhang

Last updated by author(s): Mar 29, 2024

Reporting Summary

Nature Portfolio wishes to improve the reproducibility of the work that we publish. This form provides structure for consistency and transparency in reporting. For further information on Nature Portfolio policies, see our Editorial Policies and the Editorial Policy Checklist.

Statistics

For all statistical analyses, confirm that the following items are present in the figure legend, table legend, main text, or Methods section.

n/a	Confirmed	
☐	☒	The exact sample size (*n*) for each experimental group/condition, given as a discrete number and unit of measurement
☐	☒	A statement on whether measurements were taken from distinct samples or whether the same sample was measured repeatedly
☐	☒	The statistical test(s) used AND whether they are one- or two-sided<br>*Only common tests should be described solely by name; describe more complex techniques in the Methods section.*
☒	☐	A description of all covariates tested
☐	☒	A description of any assumptions or corrections, such as tests of normality and adjustment for multiple comparisons
☐	☒	A full description of the statistical parameters including central tendency (e.g. means) or other basic estimates (e.g. regression coefficient) AND variation (e.g. standard deviation) or associated estimates of uncertainty (e.g. confidence intervals)
☐	☒	For null hypothesis testing, the test statistic (e.g. *F*, *t*, *r*) with confidence intervals, effect sizes, degrees of freedom and *P* value noted<br>*Give P values as exact values whenever suitable.*
☒	☐	For Bayesian analysis, information on the choice of priors and Markov chain Monte Carlo settings
☒	☐	For hierarchical and complex designs, identification of the appropriate level for tests and full reporting of outcomes
☒	☐	Estimates of effect sizes (e.g. Cohen's *d*, Pearson's *r*), indicating how they were calculated

Our web collection on statistics for biologists contains articles on many of the points above.

Software and code

Policy information about availability of computer code

Data collection	qPCR raw data were collected by QuantStudioTM Design & Analysis Software v1.4.2.<br>For Mass spectrometry analysis, protein identification and quantification were accomplished by MaxQuant 1.6.17.0.<br>Western blot images were collected by UVITEC Alliance micro Q9 system (UVITEC, Britain).<br>Masson's trichrome staining and H&E staining images were captured using a fully automatic digital pathology scanner (UNIC PRECICE 610, Suzhou, China).<br>The size distribution and concentration of sEVs were measured using Nanoparticle tracking analyzer (NTA, Particle Metrix, Germany).<br>The morphology of sEVs was examined by transmission electron microscope (TEM, H-7650C, Hitachi, Japan).<br>Fluorescence in situ hybridization (FISH) and immunofluorescence (IF) images were acquired on Nikon A1Si Laser Scanning confocal Microscope (Nikon Instruments Inc., Japan).<br>The data related to fluorescence intensity and absorbance were measured using an Spark™ Multimode Microplate Reader (Tecan, Spark 10m).
Data analysis	Prism 8 (Version 8.0.1) was used for data analysis, including calculation of the means, standard deviations and P-values of statistical test.<br>Image J software Version 1.53k is used to measure the gray value of western blot bands, Wound width, epithelium length, and count the number of cells in thetranswell migration assays.<br>circPrimer 2.0 soft was used to predict possible m6A modification sites in circCDK13.

For manuscripts utilizing custom algorithms or software that are central to the research but not yet described in published literature, software must be made available to editors and reviewers. We strongly encourage code deposition in a community repository (e.g. GitHub). See the Nature Portfolio guidelines for submitting code & software for further information.

Data

Policy information about availability of data

All manuscripts must include a data availability statement. This statement should provide the following information, where applicable:

- Accession codes, unique identifiers, or web links for publicly available datasets
- A description of any restrictions on data availability
- For clinical datasets or third party data, please ensure that the statement adheres to our policy

Source data are provided with this paper. The remaining data are available from this paper and supplementary materials or the corresponding authors upon request.

Research involving human participants, their data, or biological material

Policy information about studies with human participants or human data. See also policy information about sex, gender (identity/presentation), and sexual orientation and race, ethnicity and racism.

Reporting on sex and gender	N/A
Reporting on race, ethnicity, or other socially relevant groupings	N/A
Population characteristics	N/A
Recruitment	N/A
Ethics oversight	N/A

Note that full information on the approval of the study protocol must also be provided in the manuscript.

Field-specific reporting

Please select the one below that is the best fit for your research. If you are not sure, read the appropriate sections before making your selection.

☒ Life sciences ☐ Behavioural & social sciences ☐ Ecological, evolutionary & environmental sciences

For a reference copy of the document with all sections, see nature.com/documents/nr-reporting-summary-flat.pdf

Life sciences study design

All studies must disclose on these points even when the disclosure is negative.

Sample size	The sample size was based on our previous experimental results(Signal Transduct Target Ther. 2023;8(1):62; Bioact Mater. 2023;27:257-270) and was similar to the sample size commonly used in the field.Generally three independent replicates were done for in vitro experiments, and four to six independent biological replicates for in vivo experiments.
Data exclusions	No data was excluded from any of the analyses in this work.
Replication	For in vitro experiments, at least three biologically independent experiments were performed for all experiments unless otherwise stated. For in vivo experiments, n=6-8 mice/group mice or n=6-8 rats/group rats were used to analyze the wound healing rate. At least 3 mice (or rats) per group were used for histological analysis and immune cell analysis. This information is also given in the figure captions and Methods section.
Randomization	For all studies, samples were randomly divided into different experimental groups.
Blinding	No formal blinding was used in this study. Because all experiments were performed based on standardized protocols and blinding has no effect on the experiment results. Unbiased experimental procedure and data analysis were carried out as far as possible.

Reporting for specific materials, systems and methods

We require information from authors about some types of materials, experimental systems and methods used in many studies. Here, indicate whether each material, system or method listed is relevant to your study. If you are not sure if a list item applies to your research, read the appropriate section before selecting a response.

Materials & experimental systems

n/a	Involved in the study
[ ]	[x] Antibodies
[ ]	[x] Eukaryotic cell lines
[x]	[ ] Palaeontology and archaeology
[ ]	[x] Animals and other organisms
[x]	[ ] Clinical data
[x]	[ ] Dual use research of concern
[x]	[ ] Plants

Methods

n/a	Involved in the study
[x]	[ ] ChIP-seq
[x]	[ ] Flow cytometry
[x]	[ ] MRI-based neuroimaging

Antibodies

Antibodies used

primary antibodies:
IGF2BP3 Polyclonal antibody (ProteinTech, Cat No. 14642-1-AP, 1:2000 dilution for WB, 1:100 dilution for IF assays, Immunoprecipitation (IP): 0.5-4.0 ug for 1.0-3.0 mg of total protein lysate);
c-MYC Monoclonal antibody (ProteinTech, Cat No. 67447-1-Ig,1:5000 dilution for WB, 1:1000 dilution for IF assays);
CD44 Monoclonal antibody (ProteinTech, Cat No. 60224-1-Ig, 1:2000 dilution for WB, 1:200 dilution for IF assays);
Cyclin D1 Monoclonal antibody (ProteinTech, Cat No. 60186-1-Ig, 1:5000 dilution for WB assays);
GAPDH Monoclonal antibody (ProteinTech, Cat No. 60186-1-Ig, 1:100,000 dilution for WB assays);
Anti-beta Actin (Abcam, Cat No. ab8227, 1:1000 dilution for WB assays);
CDK13 Rabbit pAb (ABclonal, Cat No. A10258,1:1000 dilution for WB assays);
Anti-N6-methyladenosine (m6A) antibody (ProteinTech, Cat No. 68055-1-Ig, 1:1000dilution for meRIP assays)
CD63 Polyclonal antibody (ProteinTech, Cat No. 25682-1-AP, 1:1000 dilution for WB assays);
CD9 Polyclonal antibody (ProteinTech, Cat No. 20597-1-AP, 1:2000 dilution for WB assays);
TSG101 Polyclonal antibody (ProteinTech, Cat No. 28283-1-AP, 1:10,000 dilution for WB assays);
Alix Polyclonal antibody (ProteinTech, Cat No. 12422-1-AP, 1:20,000 dilution for WB assays);
Calnexin Monoclonal antibody (ProteinTech, Cat No. 66903-1-Ig, 1:10,000 dilution for WB assays);
Vimentin Monoclonal antibody (ProteinTech, Cat No. 60330-1-Ig, 1:1000 dilution for IF assays).

secondary antibodies:
Goat anti-Rabbit IgG (H+L) Cross-Adsorbed Secondary Antibody, Alexa Fluor™ 488 (Invitrogen, Cat No. A-11008, 1:1000 dilution for IF assays);
Goat anti-Mouse IgG (H+L) Highly Cross-Adsorbed Secondary Antibody, Alexa Fluor™ 594 (Invitrogen, Cat No. A-11032,1:1000 dilution for IF assays);
Goat Anti-Rabbit IgG (H+L), HRP Conjugate (Transgen, Cat No. HS101-01, 1:10,000 dilution for WB assays);
Goat Anti-Mouse IgG (H+L), HRP Conjugate (Transgen, Cat No. HS201-01, 1:10,000 dilution for WB assays).

Validation

All commercially available antibodies were validated by vendors. Validation statements are provided on the manufacture's website. We examined primary antibodies according to manuals, and got similar results with validation results on manufacturer's website or relevant citations.

primary antibodies:
-IGF2BP3 Polyclonal antibody used has been validated to be used for WB, IP, and IF assays.
(https://www.ptgcn.com/products/IGF2BP3-Antibody-14642-1-AP.htm)
-c-MYC Monoclonal antibody used has been validated to be used for WB and IF assays.
(https://www.ptgcn.com/products/MYC-Antibody-67447-1-Ig.htm)
-CD44 Monoclonal antibody used was validated for WB and IF assays.
(https://www.ptgcn.com/products/CD44-Antibody-60224-1-Ig.htm)
-Cyclin D1 Monoclonal antibody used was validated for WB assays.
(https://www.ptgcn.com/products/CCND1-Antibody-60186-1-Ig.htm)
-GAPDH Monoclonal antibody used was validated for WB assays.
(https://www.ptgcn.com/products/GAPDH-Antibody-60004-1-Ig.htm)
-Anti-beta Actin used was validated for WB assays.
(https://www.abcam.cn/products/primary-antibodies/beta-actin-antibody-ab8227.html)
-CDK13 Rabbit pAb used was validated for WB assays.
(https://abclonal.com.cn/Datasheet/Antibodies/A10258.pdf?v=1701335217)
-Anti-N6-methyladenosine (m6A) antibody used was validated for MeRIP assays.
(https://www.ptgcn.com/products/m6A-Antibody-68055-1-Ig.htm)
-CD63 Polyclonal antibody used was validated for WB assays.
(https://www.ptgcn.com/products/CD63-Antibody-25682-1-AP.htm)
-CD9 Polyclonal antibody used was validated for WB assays.
(https://www.ptgcn.com/products/CD9-Antibody-20597-1-AP.htm)
-TSG101 Polyclonal antibody used was validated for WB assays.
(https://www.ptgcn.com/products/TSG101-Antibody-28283-1-AP.htm)
-Alix Polyclonal antibody used was validated for WB assays.
(https://www.ptgcn.com/products/PDCD6IP-Antibody-12422-1-AP.htm)
-Calnexin Monoclonal antibody used was validated for WB assays.
(https://www.ptgcn.com/products/Calnexin-Antibody-66903-1-Ig.htm)
-Vimentin Monoclonal antibody was validated for IF assays.
(https://www.ptgcn.com/products/Vimentin-Antibody-60330-1-Ig.htm)

secondary antibodies:
-Goat anti-Rabbit IgG (H+L) Cross-Adsorbed Secondary Antibody, Alexa Fluor™ 488 was validated for IF assays. (https://www.thermofisher.cn/cn/zh/antibody/product/Goat-anti-Rabbit-IgG-H-L-Cross-Adsorbed-Secondary-Antibody-Polyclonal/A-11008)
-Goat anti-Mouse IgG (H+L) Highly Cross-Adsorbed Secondary Antibody, Alexa Fluor™ 594 was validated for IF assays. (https://www.thermofisher.cn/cn/zh/antibody/product/Goat-anti-Mouse-IgG-H-L-Highly-Cross-Adsorbed-Secondary-Antibody-Polyclonal/A-11032)
-Goat Anti-Rabbit IgG (H+L), HRP Conjugate used was validated for WB assays. (https://www.transgen.com/antibody_second/397.html)
-Goat Anti-Mouse IgG (H+L), HRP Conjugate used was validated for WB assays. (https://www.transgen.com/antibody_second/403.html)

Eukaryotic cell lines

Policy information about cell lines and Sex and Gender in Research

Cell line source(s)	human dermal fibroblasts (HDFs,Chinese Academy of Sciences' cell bank); human epidermal keratinocytes (HEKs,Chinese Academy of Sciences' cell bank).
Authentication	Cells were authenticated by STR profiling.
Mycoplasma contamination	Cell lines were tested negative for mycoplasma contamination at the time of purchase. The cell line did not exhibit any abnormal features during subsequent culture.
Commonly misidentified lines (See ICLAC register)	No commonly misidentified cell lines were used.

Animals and other research organisms

Policy information about studies involving animals; ARRIVE guidelines recommended for reporting animal research, and Sex and Gender in Research

Laboratory animals	Male diabetic mice (BKS-Leprem2Cd479/Gpt, db/db, 10-12 weeks of age) purchased from Jiangsu Jicui Yaokang Biotechnology Co., Ltd. (NanJing, China), male SD rats(6-8 weeks of age) purchased from SiPeiFu biotechnology Co., Ltd (Beijing, China), and all rodents were housed in a suitable environment with 25 °C, 50%~45% humidity, and 12 h dark/light cycle, given free access to water and food.
Wild animals	The study did not involve wild animals.
Reporting on sex	Male mice or rats were used in this study.
Field-collected samples	The study did not involve samples collected from the field.
Ethics oversight	Experimental procedures were approved by the Institutional Animal Care and Use Committee of Chinese PLA General Hospital and performed in accordance with the Animal Research: Reporting of In Vivo Experiments (ARRIVE) guidelines.

Note that full information on the approval of the study protocol must also be provided in the manuscript.

Plants

Seed stocks	n/a
Novel plant genotypes	n/a
Authentication	n/a
